# The role of IL-6 in the radiation response of prostate cancer

**DOI:** 10.1186/1748-717X-8-159

**Published:** 2013-06-27

**Authors:** Chun-Te Wu, Miao-Fen Chen, Wen-Cheng Chen, Ching-Chuan Hsieh

**Affiliations:** 1 Department of Urology, Chang Gung Memorial Hospital, Keelung, Taiwan; 2 Chang Gung University, College of Medicine, Taoyuan, Taiwan; 3 Department of Radiation Oncology, Chang Gung Memorial Hospital, Chiayi, Taiwan; 4 General Surgery, Chang Gung Memorial Hospital, Chiayi, Taiwan

**Keywords:** IL-6, Irradiation, Prostate cancer

## Abstract

**Background:**

Hormone-resistant (HR) prostate cancers are highly aggressive and respond poorly to treatment. IL-6/STAT3 signaling has been identified to link with the transition of HR and aggressive tumor behavior. The role of IL-6 in the radiation response of prostate cancer was investigated in the present study.

**Material and methods:**

The murine prostate cancer cell line (TRAMP-C1) and the hormone-resistant cell sub-line, TRAMP-HR, were used to assess the radiation response using *in vitro* clonogenic assays and tumor growth delay *in vivo*. Biological changes following irradiation were investigated by means of experimental manipulation of IL-6 signaling. Correlations among IL-6 levels, tumor regrowth, angiogenesis and myeloid-derived suppressor cell (MDSC) recruitment were examined in an animal model.

**Results:**

HR prostate cancer cells had a higher expression of IL-6 and more activated STAT3, compared to TRAMP-C1 cells. HR prostate cancer cells had a greater capacity to scavenge reactive oxygen species, suffered less apoptosis, and subsequently were more likely to survive after irradiation. Moreover, IL-6 expression was positively linked to irradiation and radiation resistance. IL-6 inhibition enhanced the radiation sensitivity of prostate cancer, which was associated with increased p53, RT-induced ROS and oxidative DNA damage. Furthermore, when mice were irradiated with a sub-lethal dose, inhibition of IL-6 protein expression attenuated angiogenesis, MDSC recruitment, and decreased tumor regrowth.

**Conclusion:**

These data demonstrate that IL-6 is important in the biological sequelae following irradiation. Therefore, treatment with concurrent IL-6 inhibition is a potential therapeutic strategy for increasing the radiation response of prostate cancer.

## Introduction

Radiation therapy (RT) is an important treatment modality for localized prostate cancer and leads to improved local control and disease-free survival of prostate cancer patients. Despite progress in the delivery mode, loco-regional post-radiotherapy relapse still occurs in a fraction of treated patients. Primarily the number of clonogens, their intrinsic cellular radioresistance and microenvironment factors determine the probability of local tumor control following radiation therapy. In the clinic, HR cancers usually appear highly aggressive and responding poorly to treatment. Several mechanisms are implicated in prostate cancer progression and androgen-independent growth [1,2]. Therefore, an improved understanding of the biological changes underlying the radiation response of prostate cancer is an important issue.

In addition to directly damaging DNA [3], the radiation-induced response is dynamic and involves several mediators and immune responses [4]. Inflammation provides a favorable environment for cancer formation and progression has been widely accepted [5]. However, there is not to detail the fundamental relationship between tumor responses to radiation and inflammation. Several inflammatory cytokines are believed to play key roles in radiation tolerance, and lead to tumor promotion, invasion, and angiogenesis [6]. Irradiation induces up-regulation of the levels of a variety of cytokines including IL-6, IL-8 and TNF-alpha. IL-6, a multi-functional cytokine, is the main activator of STAT3 signaling and the inducer for androgen receptor (AR) expression [7,8], STAT3 activation has been reported to mediate the radioresistance of tumor [9], and be up regulated by IL-6 [10]. Moreover, IL-6 has been reported to increase in a variety of tumors, and contributes to aggressive tumor growth and resistance to treatment [11-13]. In patients with high IL-6 concentrations, the response to treatment to chemotherapy and hormone therapy was worse. Patients with higher IL-6 levels have a shorter survival while a reduction in the level of IL-6 was visible in patients who responded better to therapy [14-16]. Furthermore, activated IL-6/STAT signaling is arguably the main pathway that regulates expansion of myeloid-derived suppressor cells (MDSC) [17]. The increase in MDSC induced by local irradiation was reported to facilitate tumor regrowth after irradiation [18]. Thus, IL-6 might represent a promising therapeutic target for preventing radioresistance of cancer. We reported previously that overexpressed IL-6 and activated STAT3 signaling are critical for aggressive tumor behavior and the transition of HR prostate cancer [19,20]. However, the role of IL-6 in the radiation response of prostate cancer remains unclear. In the present study, we performed cell-culture and immunocompetent animal experiments to determine the correlation between IL-6 and the biological consequences following irradiation including tumor regrowth, angiogenesis and MDSC recruitment. This investigation of the role of IL-6 in prostate cancer might lead to new strategies of enhancing the radiation response of prostate cancer.

## Materials and methods

### Cell culture and reagents

The TRAMP-C1 cell line, derived from the prostate tumor of a PB-Tag C57BL/6 (the transgenic adenocarcinoma of the mouse prostate (TRAMP)) mouse, was cultured in DMEM and 10 nM dehydroisoandrosterone. TRAMP-C1 cells were serially passaged in androgen deprived medium to establish androgen-independent growth as TRAMP-HR. TRAMP-C1 and TRAMP-HR cells were stably transfected with IL-6 silencing or control vectors. We also cultured 22RV1-HR and LNCaP- HR cells, as described earlier [21]. Mice recombinant IL-6 and IL-6 antibody were purchased from R&D (Minneapolis, MN). Stable IL-6–silenced cancer cells were generated by transfecting TRAMP-C1 and TRAMP-HR cells with the IL-6–silencing vector (IL-6 shRNA lentiviral transduction Particles in the PLKO.1 vector backbone with puromycin resistance) and selected by culturing in medium containing puromycin for 4 weeks.

### Tumor models (ectopic and orthotopic) in mice and radiation

Eight week-old male C57BL/6 J mice were used as the tumor implantation model, with the approval of the Experimental Animal Committee of our hospital. In the ectopic tumor implantation model, TRAMP-C1 and TRAMP-HR transfectants (1×10^6^ cells per implantation, five animals per group) were s.c. implanted into the dorsal gluteal region. Radiosensitivities were indicated by growth delay (*i.e.*, after irradiation, the time required for the tumor to recover to its previous volume). In the orthotopic tumor implantation model, TRAMP-C1 and TRAMP-HR transfectants (1×10^6^ cells per implantation) were intraoperatively implanted into the lateral region of the prostate gland. The extent of orthotopic tumor invasion was measured 3 weeks after implantation. The assessment of the tumor invasion ability *in vivo* was to evaluate if the implanted orthotopic tumor develope extraprostatic extension including tumor fixed or invading adjacent structures). To investigate the effect of IL-6 on tumor regrowth in tissue pre-irradiation, mice were irradiated for 10-Gy to the lower abdomen and extremities (including the prostate glands) 2 days before tumor implantation. The details are provided in the Additional file 1: Supplementary methods.

### Clonogenic assays

To determine intrinsic cellular radiosensitivities, clonogenic assays were performed. Exponentially growing cells were irradiated and incubated for 10 days at 37°C. The plates were stained with crystal violet (Sigma) to aid colony counting. Colonies containing >50 cells were scored to determine plating efficiency and the fractions of the cells surviving after application of the various treatments.

### MDSC flow cytometric analyses

MDSC are characterized by the co-expression of the myeloid-cell lineage differentiation antigens GR1 and CD11b [17]. Therefore, we used specific anti-Gr1 antibody, which reacts with a common epitope on Ly-6G and Ly-6C, and an antibody specific for CD11b (BD Pharmingen) to define mouse MDSCs as CD11b + Gr1+. We performed FACS and immunofluorescence analyses to examine the effect of irradiation on MDSC recruitment 48 h after mice received irradiation as we previously reported [22]. FACS was carried out on single cell suspensions prepared from whole tumors and spleen after digestion [23,24] and immunostaining for CD11b and GR1 with fluorescence-labeled monoclonal antibodies (BD PharMingen). The percentage of MDSC was measured by multicolor flowcytometry with the abovementioned monoclonal antibodies. Isotype-specific antibodies were used as negative controls in FACS.

### Immunohistochemical staining and immunofluorescence for tissue specimens

Formalin-fixed, paraffin-embedded tissues were cut into 5-μm sections and mounted on slides for immunohistochemical (IHC) staining. Frozen tissue specimens were cut into 5- to 8-μm cryostat sections, and incubated at room temperature for immunofluorescence staining. The details are provided in the Additional file 1: Supplementary methods.

### Intracellular free radical generation

2’7’-dichlorofluorescein diacetate (DCFH-DA) is an indicator of intracellular H_2_O_2_ and free radical levels [25]. The levels of intracellular reactive oxygen species (ROS) and oxidative DNA damage were examined after 30-min irradiation. The details are provided in the Additional file 1: Supplementary methods.

### Enzyme-linked immunosorbent assay (ELISA) of IL-6 in vitro and in vivo

IL-6 levels in culture supernatants and murine serum samples were assessed using a Mouse IL-6 Quantikine ELISA Kit (R&D Systems). The details are provided in the Additional file 1: Supplementary methods.

### Immunoblotting and statistical analysis

The details are provided in the Additional file 1: Supplementary methods.

## Results

### Response to radiation treatment

**Figure 1:**
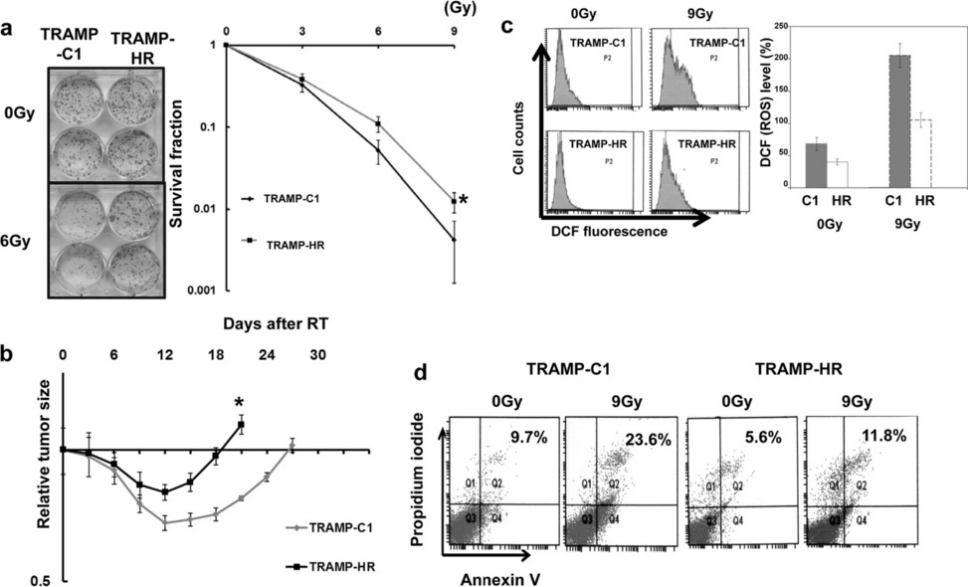
**Radiation sensitivities of hormone-sensitive and HR prostate cancer. (a)** Cells (TRAMP-C1 and TRAMP-HR) were irradiated with 0, 3, 6, or 9 Gy, and the survival curves were determined by colony-formation assay. The survival fraction was determined by enumerating colonies after irradiation exposure divided by the plating efficiency; **(b)** Tumor growth delay of irradiated ectopic tumors. The radiosensitivity is shown as growth delay after 15-Gy irradiation. HR tumors appeared more radioresistant, as shown by the shorter growth delay. Y-axis shows the ratio of tumor volume at each time point divided by that at irradiation respectively. Each point is the mean of three independent experiments; bars, SD. *, *P* < 0.05. **(c)** The intracellular ROS level was measured, using the fluorescent dye DCFH-DA, in prostate cancer cells either untreated or 30 min after 9-Gy irradiation. Y-axis represents the relative level, normalized to the level of ROS in TRAMP-C1 under control conditions. **(d)** Flow cytometric analysis using Annexin V staining for apoptosis in prostate cancer cells, either without irradiation or 24 h after 9-Gy irradiation.

Prostate cancer cells were exposed to single radiation doses of 0, 3, 6 or 9 Gy and their survival determined by colony formation assays. Figure 1a shows that HR cells had significantly greater radio-resistance compared to TRAMP-C1 cells. To determine radiation sensitivity *in vivo*, 15-Gy irradiation was performed when ectopic tumors in mice grew to a size of 0.5 cm^3^. The results show that TRAMP-C1 subcutaneous tumors had a longer-duration tumor growth delay after irradiation compared to TRAMP-HR (Figure 1b). As ROS are thought to be important mediators of radiation damage, we assayed intracellular ROS in TRAMP-C1 and HR cells 30 min after 9-Gy irradiation. Intracellular ROS levels were lower in HR than in control cells with or without irradiation (Figure 1c). Moreover, HR prostate cancer cells exhibited less RT-induced cell death compared to TRAMP-C1 cells, as revealed by annexin V and PI staining (Figure 1d).

### The role of IL-6 in the radiation sensitivity of prostate cancer

**Figure 2:**
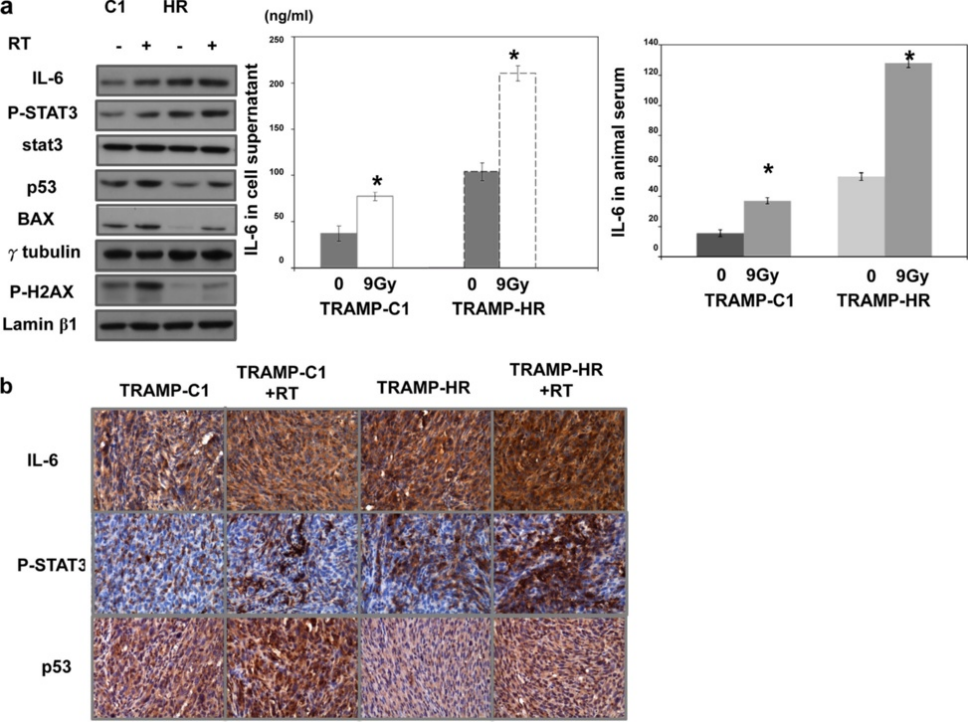
**Effects of irradiation on IL-6 expression.** IL-6 and p-STAT3 levels were evaluated by **(a)** Western blotting and ELISA 6 h after 9-Gy irradiation, and **(b)** IHC staining of ectopic tumors 48 h after 15-Gy irradiation *in vivo*. Representative slides and quantitative data are shown.

**Figure 3:**
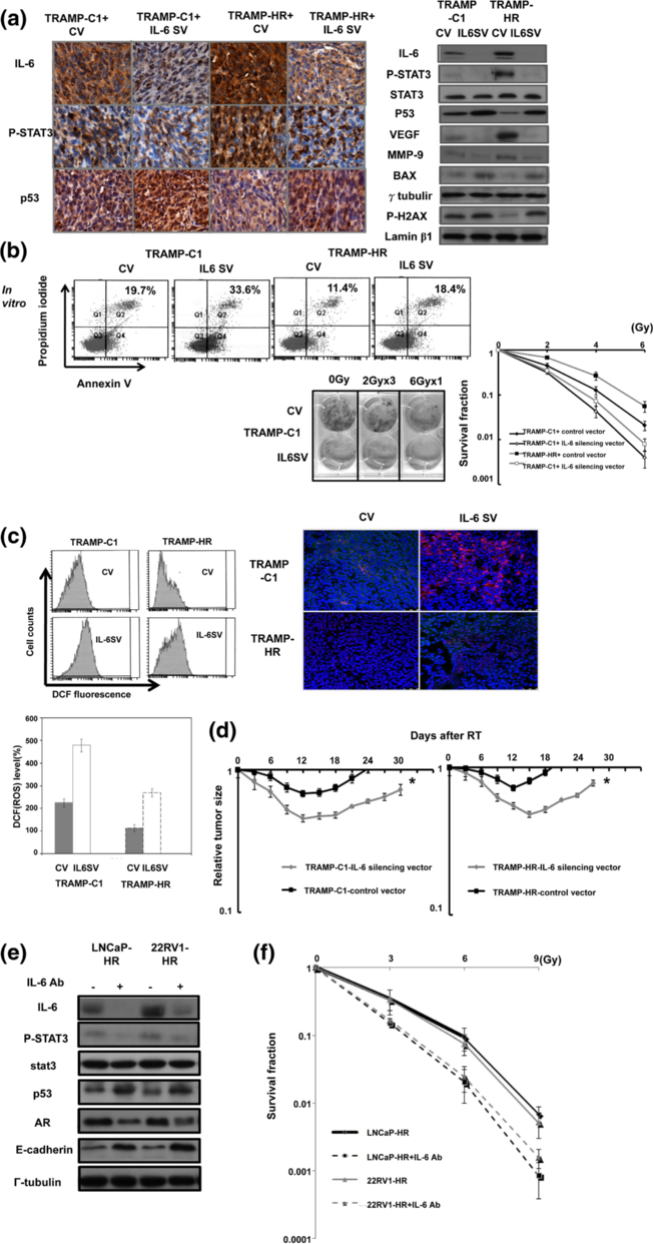
**Effects of IL-6 on the radiosensitivity of prostate cancer.** The IL-6–silencing vector significantly decreased IL-6 expression in tumor cells after irradiation, as demonstrated by Western blotting *in vitro*, IHC *in vivo***(a)**. The effects of IL-6 on *in vitro* radiosensitivity were evaluated using irradiated prostate cancer cells treated with vectors or IL-6–silencing vectors; **(b)** FACS with PI and Annexin V staining 24 h after 9-Gy irradiation and clonogenic assays; **(c)** Intracellular ROS levels as measured by the fluorescent dye DCFH-DA in prostate cancer cells, and immunofluorescence staining of oxidative DNA damage (DAPI, blue; 8-oxoG, red), 30 min after irradiation. Columns show the mean of three independent experiments; bars, SD. *, *P* < 0.05. **(d)** Effects of IL-6 on *in vivo* radiosensitivity were evaluated by means of the growth delay of ectopic prostate tumors following 15-Gy irradiation. Y-axis shows the tumor volume ratio at each time point, divided by that at the time of irradiation. (CV = cells transfected with control vectors; IL-6 SV = cells transfected with IL-6 silencing vectors). The effects of IL-6 on the expressions of activated STAT3, p53 and AR **(e)** and the sensitivity of radiation **(f)** were assessed using cells pre-incubated in the presence or absence of 5 ug/ml IL-6 neutralizing antibody for 24 h.

HR prostate cancer (LNCaP-HR and TRAMP-HR) had higher levels of IL-6, more activated STAT3 and AR compared to hormone sensitive prostate cancer cells (LNCaP and TRAMP-C1) shown in our previous and the present study [19]. We further examined the effect of irradiation on IL-6/STAT3 signaling in prostate cancer in the present study. Our data revealed that irradiation enhanced IL-6 expression by Western blotting and ELISA assay (Figure 2a), and IHC data from the irradiated ectopic tumors (Figure 2b) confirmed the *in vitro* findings. To determine the role of IL-6 in the radiosensitivity of prostate cancer, TRAMP-C1 and TRAMP-HR were transfected with IL-6-silencing or control vectors. The IL-6 silencing vector significantly inhibited IL-6 expression associated with decreased p-STAT3 (Figure 3a, Additional file 2: Figure S1). As shown in Figure 3a-c, blocking of IL-6 increased the RT-induced loss of clonogenic cells, which was associated with increased p53, apoptosis, intracellular ROS, and oxidative DNA damage. *In vivo,* IL-6 inhibition significantly prolonged the delay in prostate tumor growth after irradiation (Figure 3d). Furthermore, as shown in Figure 3e-f, IL-6 also possessed a significant impact on the radiation sensitivity of human LNCaP-HR and 22RV1-HR prostate cancer cells.

### Tumor regrowth following irradiation

**Figure 4:**
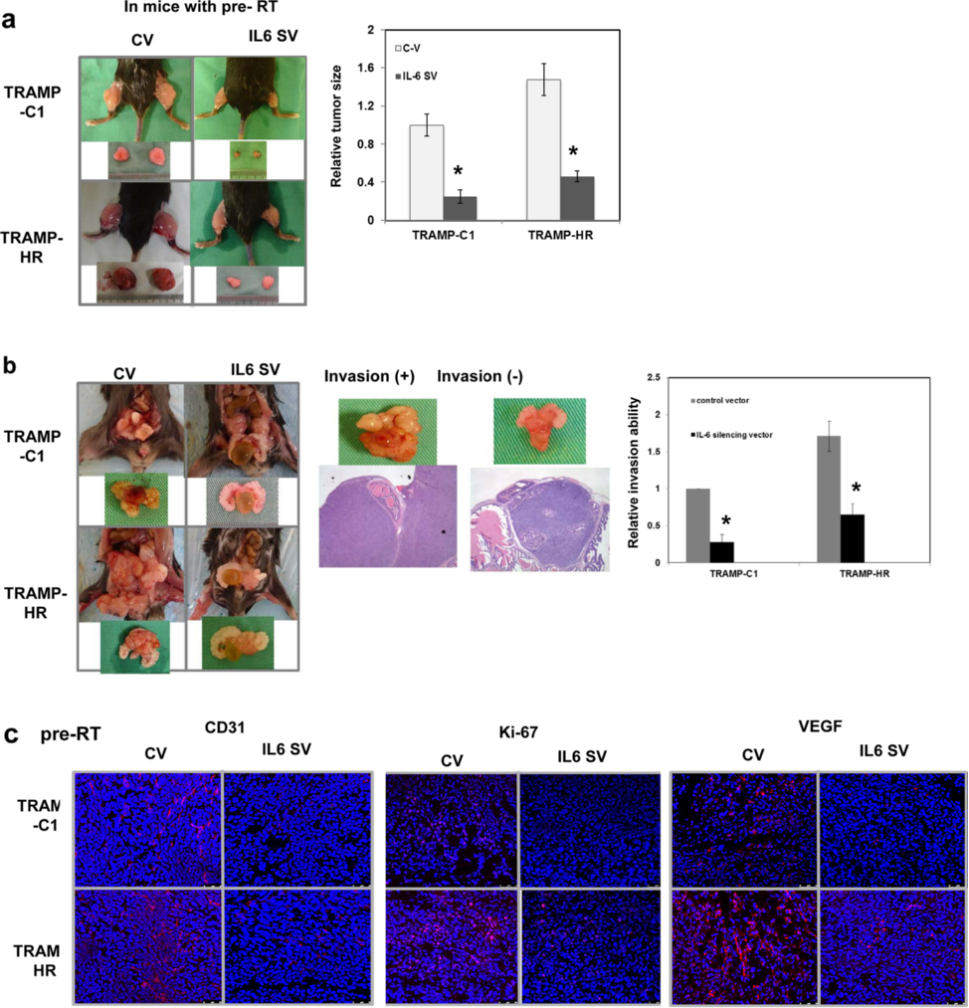
**Tumor growth in mice following 10-Gy irradiation.** The effect of IL-6 silencing vectors on tumor regrowth as demonstrated by **(a)** ectopic tumor growth curves and representative images 12 days after tumor implantation in pre-irradiated mice. **(b)** Effect of the IL-6–silencing vector on invasion was evaluated using the orthotopic tumor model. Representative images and quantitative data are shown. Y-axis represents the relative ratio, normalized to the number of TRAMP-C1 tumors developing extraprostatic extension 3 weeks after implantation in pre-irradiated mice.*, *P* < 0.05. **(c)** Expression levels of Ki-67, CD31, and VEGF were evaluated by immunofluorescence in growing tumors 12 days after implantation in irradiated mice.

As shown *in vivo*, irradiation causes tumor inhibition, but tumor regrowth after irradiation remains an important issue. To investigate this, we pre-irradiated extremity tissue and the prostate gland of mice with a 10-Gy dose before implanting prostate tumor cells to mimic tumor regrowth after irradiation. HR exhibited more rapid tumor growth and a higher ability to invade the surrounding tissues as assessed by ectopic and orthotopic tumors in pre-irradiated mice (Figure 4a-b). Angiogenesis has been reported to promote tumor progression, and CD31-mediated endothelial cell–cell interactions play a role in this process [26]. Furthermore, the Ki-67 protein is a cellular marker of proliferation. Figure 4c and Additional file 2: Figure S2, as determined using Ki-67, CD31 and VEGF immunostaining, showed that HR tumor growth in pre-irradiated tissue was associated with increased cell proliferation and angiogenesis compared with the TRAMP-C1 tumor. Furthermore, IL-6–silencing vectors significantly inhibited TRAMP-C1 and HR tumor regrowth and invasion, which was associated with attenuated expressions of angiogenic factors and STAT3 activation. Therefore, we suggest that IL-6 inhibition attenuates tumor regrowth after irradiation, an effect associated with decreased cell proliferation and attenuation of angiogenesis.

### Effect of IL-6 on MDSC induction

**Figure 5:**
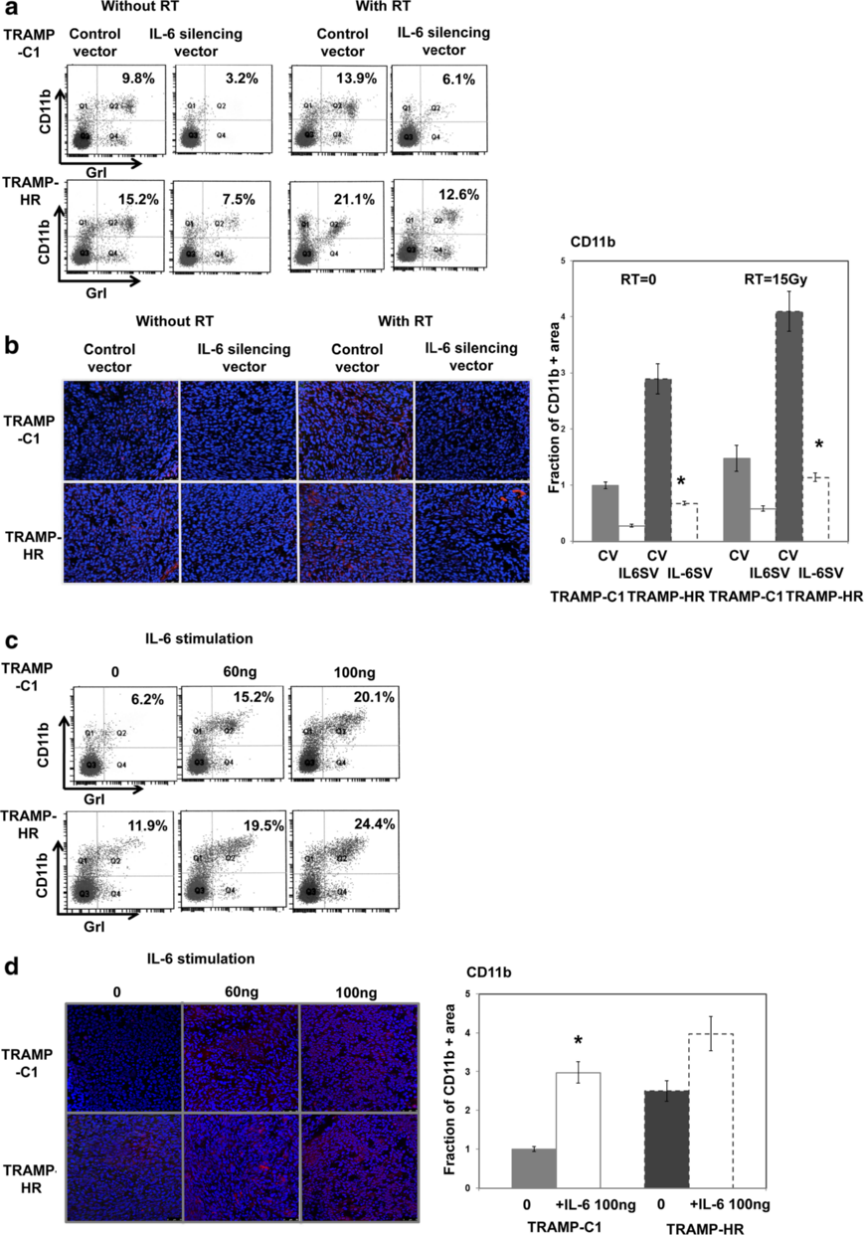
**IL-6 is linked to MDSC recruitment.** Effects of IL-6 and irradiation on MDSC recruitment in tumors were evaluated by **(a)** FACS of spleen using GrI-CD11b staining, and **(b)** immunofluorescence of tumor (DAPI, blue; CD11b, red) in mice bearing tumors (tumor size 300 ~ 350 mm3) with or without IL-6–silencing vectors or 48 h after 15-Gy irradiation or sham-irradiation. Representative images are shown. Quantification of CD11b expression was calculated as the number of cells positive for CD11b immunofluorescence divided by the total number of cells for each condition. Y-axis represents the ratio normalized to the value of TRAMP-C1 cells. Columns are the means of three independent experiments; bars, SD. *, *P* < 0.05. The effect of IL-6 on MDSC recruitment was further evaluated by **(c)** FACS of the spleen and **(d)** immunofluorescence of tumor specimens in pre-irradiated mice bearing tumors (with or without IL-6 stimulation) for 2 weeks.

We reported previously that MDSC contributes to tumor promotion in IL-6–positive prostate cancer, at least in part. Moreover, the increase in MDSC induced by local irradiation facilitates tumor regrowth after irradiation [18]. Therefore, we examined the correlation between IL-6 and MDSC recruitment after irradiation by analyzing MDSC levels in tumor-bearing mice 48 h after irradiation [10,22], with or without IL-6-silencing vectors. The recruitment of CD11b + Gr1+ myeloid cells increased significantly in mice bearing a HR tumor, compared to that in TRAMP-C1 tumor, by FACS and immunofluorescence analyses, and recruitment of CD11b + Gr1+ cells to tumors were augmented by irradiation (Figure 5a-b). Furthermore, the IL-6 silencing vector significantly abrogated the recruitment of MDSC with or without irradiation. To determine further whether MDSC accumulation was driven by IL-6 stimulation, we examined MDSC levels in tumors in pre-irradiated mice with or without IL-6 stimulation. As determined by FACS and immunofluorescence, IL-6 stimulation increased MDSC accumulation (Figure 5c-d). These results suggested that IL-6 plays an important role in the induction of MDSC recruitment, which might subsequently contribute to the increased invasiveness of tumors that regrow after irradiation.

## Discussion

The transgenic adenocarcinoma of the mouse prostate (TRAMP) model closely mirrors the pathogenesis of human prostate cancer. TRAMP-C1 is a prostate cell line derived from a TRAMP tumor and widely used to assess novel therapeutic approaches for prostate cancer. To evaluate the radiation response in an immunocompetent model, a TRAMP-C1 prostate cancer cell line, derived from adenocarcinoma of the mouse prostate, and TRAMP-HR, derived from TRAMP-C1 cells cultured in androgen-deprived medium, were used in the present study. The radiation response was evaluated using assays that take into account various types of radiation-induced cell death, specifically, *in vitro* clonogenic assays and *in vivo* tumor size measurements. As determined by clonogenic assay and tumor-growth delay in mice, HR cells are more resistant to irradiation compared to TRAMP-C1 tumor cells. Ionizing radiation can induce various cell-death processes, and the biological effects of RT are mediated largely by reactive oxygen intermediates [27]. In this study, elevated RT-induced apoptosis and a greater increase in ROS were observed in TRAMP-C1 cells compared with HR cells. It was reported that IL-6 contributed to the conversion of prostate cancer to an androgen-independent state in xenografts models [8,28], and anti-IL-6 monoclonal antibody have been applied to treat metastatic castration-resistant prostate cancer in clinical studies [29]. Our data also showed that overexpressed IL-6 had significant impact on androgen- independent growth and aggressive behavior of prostate cancer. In prostate cancer, although the critical role of IL-6 in carcinogenesis has been highlighted [19,28], its role in the radiation response of prostate cancer requires further investigation. IL-6 is an important transcription factor that regulates oncogenic signaling and induces STAT3 activation [30,31] in a variety of malignancies by promoting proliferation and inhibiting apoptosis. Moreover, IL-6 has also been correlated with cancer treatment resistance where modulating the IL-6 pathway directly affects the cellular resistance to treatment, including breast cancer, ovarian cancer and esophageal cancer [14-16]. In the present study, we examined the role of IL-6 in the response to radiation and tumor regrowth following radiation in prostate cancer. Mechanisms underlying DNA repair and the DNA damage response (DDR) play key roles in the regulation of radiation-induced cell death [32]. IL-6 is reported play a role in DRR to suppress cell death induced by treatment. IL-6 increases the expression of several antiapoptotic proteins through STAT3 [13]. Our data indicate that IL-6 expression was linked with irradiation and the biological changes following irradiation for prostate cancer. When IL-6 was inhibited by the silencing vector, increased RT-induced cell death associated with increased RT-induced ROS and oxidative DNA damage was observed. Moreover, IL-6 inhibition was shown to decrease the level of p-STAT3 and increase those of p53 and p-H2AX in both TRAMP-C1 and HR cancer cells after irradiation. We found that a significant decrease in the expression of p53 was noted in HR cells, and associated with radiation resistance [21]. P53 has been reported to play an important role in ROS scavenging and DNA repair, and may contribute to the increased cell death and radiosensitivity [21,33]. Moreover, several studies revealed that the reduced p53 could induce prostate cancer cells to become androgen unresponsive and decrease the apoptosis induced by androgen deprivation [34,35]. Activated STAT3 was reported to bind to p53 and represses its function as a regulator of apoptosis [36]. Accordingly, we suggest that the decreased STAT3 activation and ROS scavenging ability associated with increased apoptosis and p53 levels contributes to the IL-6–silencing-vector induced radiosensitization in prostate cancer, at least in part.

Radiation therapy is widely used to achieve locoregional control of cancers. Unfortunately, the initial tumor response is often followed by a relapse. For decades, studies have focused on the radiosensitivity of cancer cells. It is becoming increasingly clear that multiple changes in tumor stroma may also determine the treatment outcome. Tumor regrowth after radiotherapy may be substantially affected by systemic factors. The gold standard for assessment of the clinical efficacy of a particular treatment *in vivo* is the long-term response of tumors using mouse models. Tumor responsiveness to treatment is influenced both directly and indirectly by the vasculature and tumor cell proliferation. To investigate the mechanisms underlying tumor regrowth in prostate cancer, we pre-irradiated the prostate and lower extremities of mice with a single 10-Gy dose before implanting prostate cancer cells to mimic local relapse after radiotherapy. IL-6/STAT3 signaling was reported to stimulating tumor invasion, EMT changes, and promote the surviving tumor cells after therapy to acquire treatment resistance [37] via microenvironment- mediated resistance. Increased IL-6 in LNCaP-IL-6+ cancer cells showed a growth advantage, STAT3 activation, high expression of VEGF association with androgen-independent growth [38,39]. In the present study, tumor growth combined with IF and IHC data using VEGF, Ki-67, and CD31 staining, suggest that the IL-6–silencing vector slowed tumor regrowth and attenuated the aggressive behavior of the orthotopic tumor model, which were associated with decreased STAT3 activation, cell proliferation and angiogenesis following irradiation. Therefore, in addition to increased cell death, slower tumor regrowth associated with attenuated angiogenesis may contribute, in part, to the radiosensitization induced by IL-6 inhibition *in vivo*.

Ionization radiation intensifies the recruitment of distal stroma, in the form of inflammatory bone-marrow-derived cells, to the tumor and its surroundings. Furthermore, relapses within a pre-irradiated area are associated with an increased risk of local invasion and metastasis formation in several cancers. MDSC are an important subset of cells that contribute to an immunosuppressive tumor microenvironment, and are reported to facilitate tumor regrowth after irradiation [17,40]. We reported previously that MDSCs might be responsible for tumor promotion by IL-6 in prostate cancer. Moreover, we identified a positive correlation between circulating IL-6 level and tumor regrowth in irradiated mice with prostate cancer. Therefore, we further examined the link between MDSC and circulating IL-6 in tumor relapse post-irradiation. Irradiation increased MDSC recruitment in tumor-bearing mice. Furthermore, there was a positive correlation between the MDSC subpopulation and IL-6 levels in the mouse tumors. IL-6–silencing vectors significantly reduced MDSC infiltration, and IL-6 stimulation had a positive impact on MDSC recruitment. These findings suggest that IL-6 plays a critical role in MDSC recruitment and tumor regrowth after irradiation.

In summary, IL-6 inhibition sensitizes tumor cells to irradiation, increasing cell death and DNA damage, and mitigates tumor regrowth after irradiation by eliminating RT-triggered MDSC infiltration and angiogenesis. Thus the IL-6 level might be significant predictor of the radiation response in prostate cancer. In future, we will further determine if targeting IL-6 is a useful strategy for sensitizing prostate cancer to irradiation.

## Competing interests

The authors confirm that there are no conflicts of interest that could be perceived as prejudicing the impartiality of the research reported.

## Authors’ contributions

CTW conceived of the study, performed the study and coordination and assisted in editing of manuscript. MFC conceived of the study and participated in its design and coordination. WCC conceived part of the study and performed the statistical analysis. CCH participated in its design and coordination. All authors read and approved the final manuscript.

## Supplementary Material

Additional file 1 Supplementary methods. Click here for file

Additional file 2 Additional Figures S1 and S2. Click here for file
