# Supplementary material for: The role of IL-6 in the radiation response of prostate cancer
Source: Radiat Oncol. 2013 Jun 27;8:159. doi: 10.1186/1748-717X-8-159 (PMC3717100; doi:10.1186/1748-717X-8-159)
Supplement: Additional file 1 — Supplementary methods. [file 1748-717X-8-159-S1.doc]

**Supplementary Methods**

*Tumor models (ectopic and orthotopic) in mice and radiation*

In the ectopic tumor implantation model, TRAMP-C1 and TRAMP-HR transfectants (1x10^6^ cells per implantation, five animals per group) were s.c. implanted into the dorsal gluteal region. Tumor size was measured every three days after implantation (day 0). The tumor volume was calculated assuming an ellipsoid shape. To determine the radiosensitivity *in vivo*, local irradiation for 15Gy was performed when ectopic tumors reached 0.5 cm^3^, and tumor size was measured every 3 days thereafter. For irradiation locally, anesthetized mice were restrained, and ectopic tumors were irradiated using 6 MV X-ray from a linear accelerator with a 1.5-cm bolus on the surface. Control mice were subjected to sham-irradiation. The relative tumor volume normalized to the tumor size at the time of irradiation determined the curves of tumor in mice exposed to irradiation. Radiosensitivities were indicated by growth delay (*i*.*e*., after irradiation, the time required for the tumor to recover its previous volume). Duplicate experiments were performed for growth delay analyses. In the orthotopic tumor implantation model, TRAMP-C1 and TRAMP-HR transfectants (1x10^6^ cells per implantation, five animals per group) were intraoperatively implanted into the lateral region of prostate gland. The extent of orthotopic tumor invasion was measured 3 weeks after implantation to check if the implanted orthotopic tumor develope extraprostatic extension including tumor fixed or invading adjacent structures). The effect of IL-6 stimulation on MDSC recruitment was also investigated *in vivo*. For the treated group, an intraperitoneal injection of IL-6 (60 or 100 ng per mouse, 3 times per week) was started one day before tumor implantation.

*Immunohistochemical staining and immunofluorescence for tissue specimens*

Formalin-fixed, paraffin-embedded tissues were cut into 5μm sections, mounted on slides, deparaffinized with xylene and dehydrated using a graded ethanol series. They were incubated overnight with antibodies against IL-6, p-STAT3, STAT3, VEGF, CD31, p53 and Ki-67 at 4^0^C. After 3 washes in PBS, the sections were incubated with biotinylated secondary antibody for 10 min and the sections were counterstained with hematoxylin. Frozen tissue specimen were cut into 5 to 8μm cryostat sections, allowed to come to room temperature, fixed in cold acetone (-20^o^C) for 10 min, and incubate for 20 min in 10% goat serum in PBS. They were incubated overnight with antibodies against IL-6, CD11b, CD31, VEGF and Ki-67 (1:20) at 4^0^C, and incubate with fluorescein or texas red-conjugated secondary antibody for 1h.

*Intracellular free radical generation*

2’7’-dichlorofluorescein diacetate (DCFH-DA) is an indicator of intracellular H2O2 and free radicals. Briefly, cells were washed with PBS and incubated in phenol red-free and serum-free medium containing 20 mM DCFH-DA for 15 min, then treated with 9 Gy irradiation; controls were untreated. The level of intracellular reactive oxygen species (ROS) and oxidative DNA damage were examined 30 min after irradiation. H2O2 oxidizes DCFH to DCF; DCF fluorescence was detected by a flow cytometer equipped with a 488 nm argon laser.

*Enzyme-linked immunosorbent assay (ELISA) for IL-6 level in vitro and in vivo*

The levels of IL-6 in the cellular supernatants and murine serum samples were analyzed using Mouse IL-6 Quantikine ELISA Kit (R&D system). To test IL-6 level in cellular supernatant, cells were cultured with 1ml serum-free medium for 24h in 6-well plates. The medium was collected and clarified by centrifugation at 3000g. For circulating IL-6 level *in vivo*, blood samples were drawn from murine heart for assay and serums were kept frozen and then thawed shortly before determination of IL-6 levels.

*Immunoblot analysis*

For Western blotting of whole cell extract, cells were treated in lysis buffer (Calbiochem, La Jolla, CA). An NE-PER kit (Pierce, Rockford, IL) was used to separate nuclear and cytoplasmic proteins. Equal amounts of protein were loaded on to SDS-PAGE gels. After electrophoresis, the proteins were transferred to nitrocellulose membranes. Antibodies specific for IL-6, VEGF, MMP-9, STAT3, pSTAT3 ^Tyr705^, bax, p-H2AX and p53 were obtained from Santa Cruz Biotechnology, Inc., Research & Diagnostics Systems, Inc. (Minneapolis, MN USA), and Cell Signaling (Danvers, MA). The membrane was reprobed with antibody r-tubulin or nuclear lamin to normalize protein loading.

*Statistical analysis*

Significance of difference between samples was determined using Student’s t-test. Data are presented as mean±standard deviation (SD). Each experiment was at least independently carried out two times, with three repeats each. A probability level of p<0.05 was adopted throughout to determine statistical significance unless otherwise stated.

**Supplementary Figure Legend**

**Figure 1** The IL-6–silencing vector significantly decreased IL-6 expression in tumor cells after irradiation, as demonstrated by ELISA assay *in vitro* and *in vivo*.

**Figure 2** The IL-6–silencing vector significantly decreased the expression levels of Ki-67 and CD31 in growing tumors 12 days after implantation in irradiated mice, as evaluated by IHC staining
