# Supplementary material for: The role of IL-6 in the radiation response of prostate cancer
Source: Radiat Oncol. 2013 Jun 27;8:159. doi: 10.1186/1748-717X-8-159 (PMC3717100; doi:10.1186/1748-717X-8-159)
Supplement: Additional file 2 — Additional Figures S1 and S2. [file 1748-717X-8-159-S2.pptx]

## Slide 1
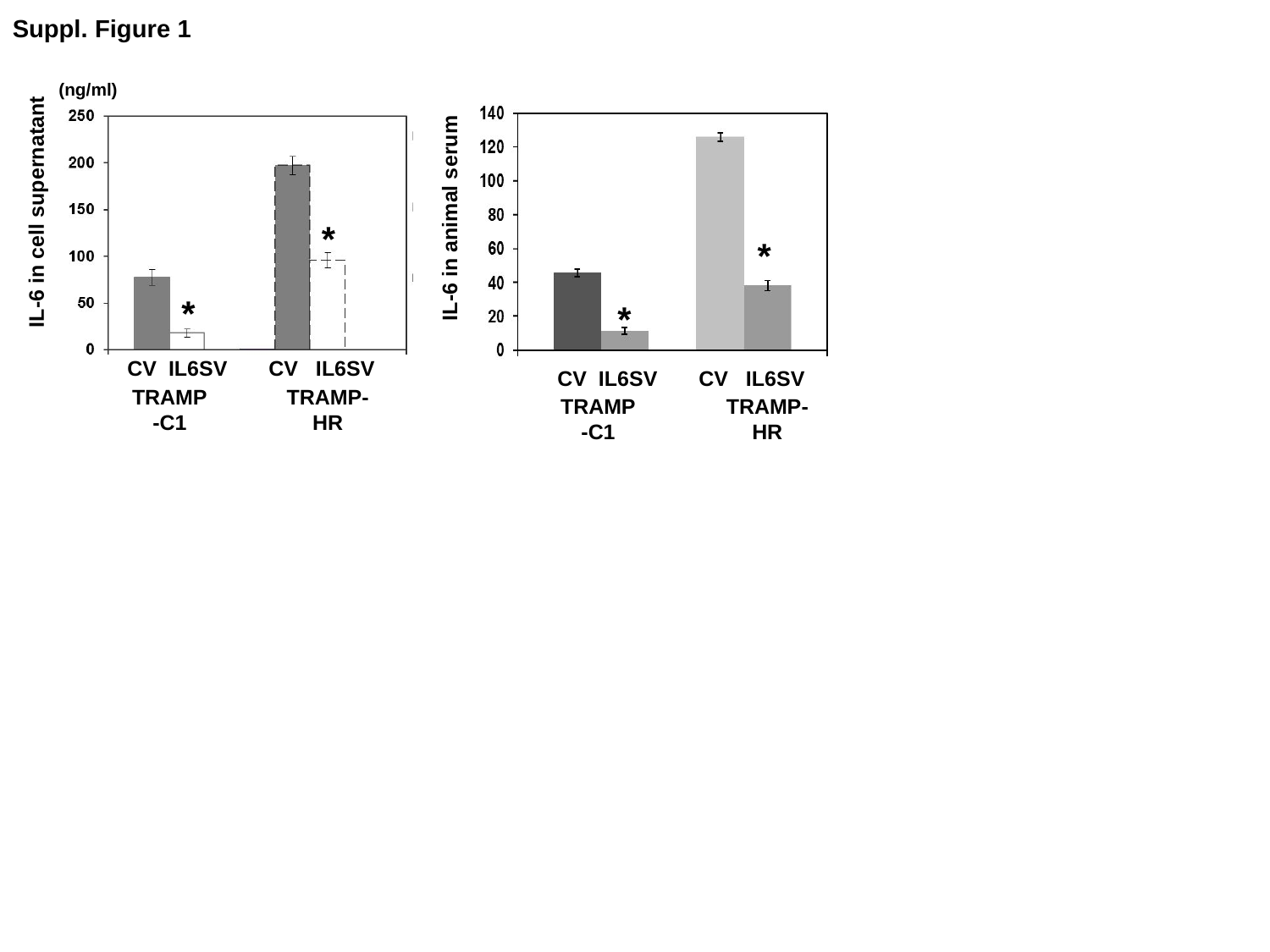

Suppl. Figure 1
(ng/ml)
 IL-6 in cell supernatant
IL-6 in animal serum
*
*
*
*
CV IL6SV CV IL6SV
CV IL6SV CV IL6SV
TRAMP-C1
TRAMP-HR
TRAMP-HR
TRAMP-C1

## Slide 2
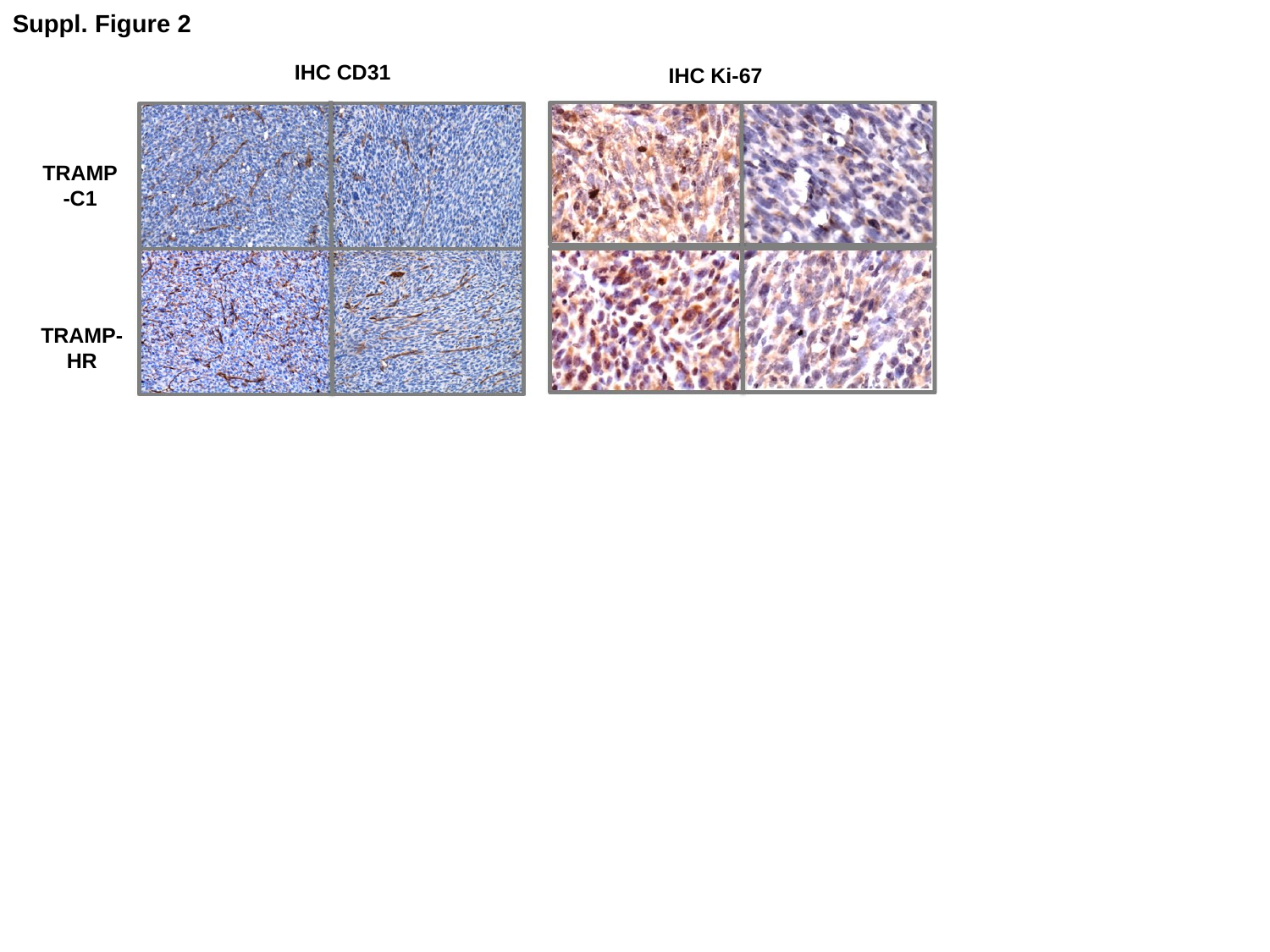

Suppl. Figure 2
IHC CD31
IHC Ki-67
TRAMP-C1
TRAMP-HR
